# Systemic lupus erythematosus associated with multiple myeloma: Two case reports and a literature review

**DOI:** 10.1002/iid3.755

**Published:** 2022-12-31

**Authors:** Li Lian, Kang Wang, Shengqian Xu

**Affiliations:** ^1^ Department of Rheumatology & Immunology The First Affiliated Hospital of Anhui Medical University Hefei Anhui China

**Keywords:** case report, multiple myeloma, renal dysfunction, systemic lupus erythematosus

## Abstract

**Introduction:**

Systemic lupus erythematosus (SLE) complicated by multiple myeloma (MM) is a relatively rare clinical presentation, and is easily ignored due to their similar or even identical manifestation, which may lead to misdiagnosis and mistreatment.

**Case report:**

We report two cases of SLE with MM. Case 1 was a 59‐year‐old male who was diagnosed with SLE 11 years ago. Abnormal kidney function was detected 4 months ago and a bone marrow aspirate revealed MM. He then received three cycles of bortezomib, dexamethasone, and chemotherapy with liposomal doxorubicin, and one cycle of lenalidomide plus dexamethasone. He died of infectious shock. Case 2 was a 58‐year‐old female who was diagnosed with SLE 27 years ago. After the onset of abnormal renal function 4 years ago, the patient was still treated according to SLE disease activity. When renal function rapidly deteriorated, serum and urine immunofixation electrophoresis was positive for IgG γ with free light chains and she was diagnosed with SLE complicated by MM. She did not agree to the treatment for MM as advised and was discharged from the hospital against medical advice. Case 2 died of cardiac failure. Thirteen cases of SLE with MM reported from 2000 to 2022 in PUBMED and Mendeley and our above two cases were reviewed. Among the 15 patients, 13 were females and 2 were males. The median age at the time of SLE with MM diagnosis was 50 years, and the median time to a delayed diagnosis was 7 years. The serum monoclonal immunoglobulin level was elevated and extramedullary manifestations of renal dysfunction were common.

**Conclusions:**

An elevated monoclonal immunoglobulin level or newly unexplained renal dysfunction occurring in a patient with SLE should prompt monitoring and further screening of MM, rather than treatment as a secondary manifestation of SLE.

## Graphical abstract

Multiple myeloma should be considered in patients with Systemic lupus erythematosus and an elevated monoclonal immunoglobulin level or newly unexplained renal dysfunction.

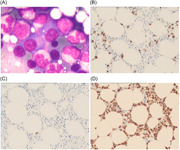

## 1 INTRODUCTION

Systemic lupus erythematosus (SLE) is an autoimmune inflammatory connective tissue disease that affects multiple organs and occurs mostly in young women. Quantitative studies have shown that genetic, endocrine, infectious, immune dysfunction, and environmental factors are related to the pathogenesis of SLE.^1^ The association between SLE and lymphoproliferative syndromes is frequently reported in the literature.^2^ Multiple myeloma (MM) is a malignant plasma cell disorder in which tumor cells originate from plasma cells in the bone marrow.^3^ B lymphocytes develop to their final functional stage as plasma cells, thus MM is designated as a plasmacytoma (i.e., a single B cell lymphoma).^3^ SLE is usually reported to be associated with different types of plasma cell disorders, such as monoclonal gammopathy of undetermined significance (MGUS) and amyloid degeneration. Some patients with MGUS progress to MM. Among SLE patients, the incidence of MGUS ranges between 2.2% and 3.3%.^4^, ^5^ Ali et al.^6^ reported that the incidence of MGUS in SLE patients is up to 5.4%, which is higher than the general population. Moreover, monoclonal IgG is the most common type.^5^ Although the incidence of MGUS is higher in SLE patients than general population, the coexistence of SLE and MM is rarely reported, and large population‐based studies are lacking. We report two cases in which MM occurred 11 and 27 years after the onset of SLE in our hospital, a large comprehensive public hospital with 4990 open beds and 5.85 million outpatient visits annually. Delayed diagnosis of co‐existing of SLE and MM need more concerns on this disease. We also analyzed the clinical data of previously published related cases to describe the clinical features of MM in SLE patients.

## 2 CASE REPORT AND LITERATURE REVIEW

### 2.1 Case report

#### 2.1.1 Case 1

A 59‐year‐old male was admitted to our hospital in May 2021 due to “joint swelling and pain with fever for 11 years, and abnormal kidney function diagnosed 4 months ago.” This patient was diagnosed with SLE in 2011 (11 years ago) with joint swelling and pain as presenting symptoms, decreased white blood cell and platelet counts, and positivity for several autoantibodies (ANA: positive; Resistance to DS‐DNA: positive). No family history of SLE, no history of serious illness and mental illness was reported by the patient. The physical examination on admission was unremarkable. The patient was given prednisone acetate (60 mg/day), which was then tapered to 5 mg/day. Hydroxychloroquine (200 mg/day) and cyclosporine (150 mg/day) were given. The health status of the patient remained stable between 2016 and 2020. He discontinued the use of cyclosporin for 4 years; however, abnormal kidney function was detected during an outpatient follow‐up visit in March 2021 with the following laboratory findings: monoclonal immunoglobulin, increased; serum creatinine (SCr), 101.0 ummol/l; blood urea nitrogen (BUN), 5.33 mmol/L; uric acid, 333 mmol/L; immunoglobulin G (IgG), 23.84 g/L; immunoglobulin A (IgA), 0.86 g/L; immunoglobulin M (IgM), 0.67 g/L; C3, 0.50 g/L; urine protein, negative; erythrocyte sedimentation rate (ESR), 70; C‐reactive protein (CRP), 80.20; white blood cell (WBC) count, 5.37 × 10^9^/L; red blood cell (RBC) count, 3.77 × 10^12^/L; hemoglobin (Hb),116 g/L; and platelet (PLT) count,165 × 10^9^/L. In consideration of SLE activity, prednisone was increased to 20 mg daily. Cyclophosphamide (CTX), 0.4 g every 2 weeks, was administered 4 times. The patient's fatigue did not improve and he was admitted to our hospital on 29 May 2021. The admission examination revealed the following: negative for urine protein; ESR, 59 mm/h; creatinine, 230 μmol/L; BUN, 15.07 mmol/L; uric acid, 677 μmol/L; IgG, 24.55 g/L (normal range, 7.60–16.60 g/L); IgA, 0.9 g/L (normal range, 0.71–3.35 g/L); IgM: 0.79 g/L (normal range, 0.48–2.12 g/L); C3, 0.5 g/L; 24‐h urine total protein, 0.27 g/d; and calcium, 1.89 mmol/L. Serum and urine immunofixation electrophoresis was positive for IgG λ with free light chains. A bone marrow aspirate revealed MM. He was transferred to the Department of Hematology and received three cycles of bortezomib, dexamethasone, and chemotherapy with liposomal doxorubicin. No bone marrow improvement was achieved after three cycles with double‐checks and review. He later received one cycle of lenalidomide plus dexamethasone but died of infectious shock on December 1, 2021. The timeline of Case 1 is shown in Table 1.

**Table 1:** Timeline of Case 1

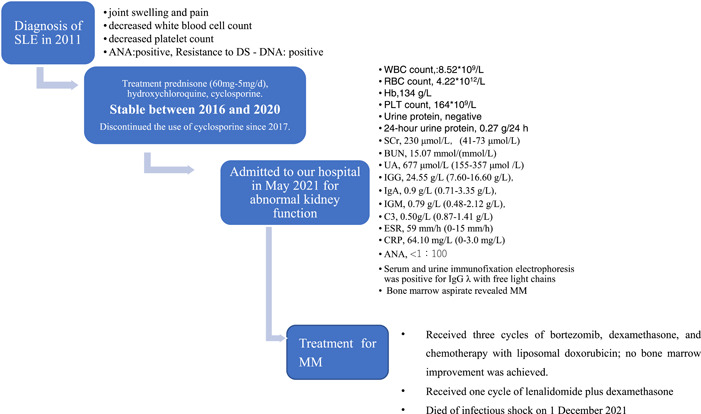

Abbreviations: MM, multiple myeloma; PLT, platelet; SLE, systemic lupus erythematosus.

#### 2.1.2 Case 2

A 58‐year‐old female was admitted to our hospital in January 2022 due to a “diagnosis of SLE for 27 years and abnormal kidney function for 4 years, with exacerbation of symptoms for 1 week.” No family history of SLE, no history of serious illness and mental illness was reported by the patient. The physical examination on admission showed the patient to be overweight with a blood pressure of 200/100 mmHg, an anemic appearance, and mild edema of the lower limbs. She was diagnosed with SLE in 1994 (27 years ago) at another hospital, with facial erythema, proteinuria, positivity for antinuclear antibody (ANA), and thrombocytopenia as presenting symptoms. She was prescribed 60 mg‐10 mg/day of CTX, mycophenolate mofetil (0.75 g bid), and hydroxychloroquine (300 mg/d) for long‐term use. After the patient was stabilized, the immunosuppressive drugs were discontinued for nearly 10 years, except for prednisone acetate (10 mg/day) plus hydroxychloroquine (300 mg/d). The patient presented with abnormal kidney function in April 2018 at another hospital, with 2–3 + urine protein. The 24‐h urine total protein was 1.06–1.43 g. No treatment was offered. The following clinical data were available in July 2019: creatinine, 175 μmol/L; BUN, 16.18 mmol/L; uric acid, 580 μmol/L; calcium and phosphorus, normal range; urine protein, 2+; and cylindruria, negative. The following clinical data were available in September 2019: creatinine, 150 μmol/L; BUN, 13.8 mmol/L; uric acid, 568 μmol/L; C3, 0.74 g/l; IgG, 22.13 g/L (normal range, 7.60–16.60 g/L); IgA, 1.51 g/L (normal range, 0.71–3.35 g/L); and IgM, 0.37 g/L (normal range, 0.48–2.12 g/L). Active SLE was considered and the patient was given mycophenolate mofetil (MMF) (1.5 g/day for 8 months). The patient had a follow‐up examination in March 2020 and the following clinical data were available: creatinine, 208 μmol/L; BUN, 17.2 mmol/L; uric acid, 557 μmol/L; and IgG, 21.09 g/L. The proteinuria did not improve on re‐testing. Active SLE was considered, thus MMF was discontinued and the treatment was changed to CTX (0.4 g every 2 weeks for 6 months) however, the urine protein still fluctuated between 2+ and 3+. The following clinical data were available in August 2020: creatinine, 287 μmol/L; BUN, 19.6 mmol/L; uric acid, 452 μmol/L; and IgG, 20.93 g/L. CTX was continued (0.4 g every 2 weeks for 6 months). The following clinical data were available in May 2021: creatinine, 388 μmol/L; BUN, 29 mmol/L; uric acid, 630 μmol/L; serum calcium, 3.33 mmol/L (elevated); and IgG, 19.87 g/L. CTX was administered for 14 months and renal function did not improve. Due to a concern of chronic renal insufficiency, the immunosuppressant was discontinued and treatment was initiated to reduce kidney damage and control blood pressure (antihypertensive drugs: calcium antagonist + vasodilator). The following clinical data were available in August 2021: creatinine, 337 μmol/L; BUN, 22.40 mmol/L; uric acid, 473 μmol/L; and IgG, 21.83 g/L. The patient was re‐examined upon admission to our hospital in December 2021, with the following laboratory findings: ANA, 1:320; and extractable nuclear antigen (ENA), negative. The previous treatment was continued.

The patient was re‐examined upon admission to our hospital in January 2022 with the following laboratory findings: creatinine, 776 μmol/L; BUN, 34.13 mmol/L; uric acid, 514 μmol/L; serum calcium, 3.5 mmol/L; IgG, 22.63 g/L; IgA 1.10 g/L; IgM, 0.25 g/L (below normal); and C3, 0.73 g/L. A diagnosis of MM was considered, and serum and urine immunofixation electrophoresis revealed positivity for IgG γ with free light chains. The diagnosis of MM was confirmed. The patient was advised to have MM treated in the Department of Hematology. She had a myocardial infarction and received treatment for myocardial infarction with aspirin and atorvastatin. The patient was very old with many underlying diseases, and her family members were concerned that the treatment was too difficult and costly with a low success rate. Therefore, the patient did not receive treatment for MM as advised. She was later discharged against medical advice. During a telephone follow‐up on 7 February, a family member reported that the patient had died on February 3, 2022 due to cardiac failure. The timeline and examination results of Case 2 are shown in Table 2.

**Table 2:** Timeline and examination results of Case 2

Year	2019‐07	2019‐09	2020‐03	2020‐08	2021‐01	2021‐05	2021‐12	Reference range
WBC	8.14	8.92	10.15	7.34	8.51	7.51	3.39	3.5–9.5 × 10^9^/L
RBC	3.55	3.33	3.08	2.77	2.67	2.59	3.07	3.80–5.10 × 10^12^/L
Hemoglobin	109	106	101	84	79	78	93	115–150 g/L
PLT	217	211	250	235	224	219	165	125–150 × 10^9^/L
SCr	175	150	208	287	337	388	776	41–73 μmol/L
BUN	16.18	13.8	17.2	19.6	22.4	29	34.13	2.6–7.50 mmol/L
UA (μmol/L)	580	568	557	452	473	630	514	155–357 μmol/L
Urine protein	2+	2+	2+	2+	2+		2+	negative
24‐h urine protein						1.06	1.43	0–0.15 g/24 h
IgG (g/L)	19.94	22.13	21.09	20.93	21.83	19.87	25.94	7.60–16.60 g/L
IgA (g/L)	1.72	1.51	1.37	1.26	1.31	0.96	1.02	0.71–3.35 g/L
IgM (g/L)	0.33	0.37	0.29	0.30	0.29	0.25	0.38	0.48–2.12 g/L
C3	0.70	0.74	0.78	0.79	0.85	0.76	0.68	0.87–1.41 g/L
C4	‐	‐	0.26	0.24	0.27	‐	0.26	0.10–0.40 g/L
ESR	27	44	33	40	82	61	45	0–20 mm/h
CRP	30	10.10	4.00	5.70	—	11.50	8.7	0–3 mg/L
ANA	1:320			1:320			1:320	<1:100
DS‐DNA	‐						13	0100 IU/L
*Treatment*								
Prednisone	5 mg/D	5 mg/d	5 mg/d	5 mg/d	5 mg/d	5 mg/d	5 mg/d	5 mg/d
Immunosupressant	MMF	MMF		CTX	CTX0.4 g			
	1.5 g/d	1.5 g/d	CTX	0.4 g	Q2w		Hemodialysis	Hemodialysis
			0.4 g	Q2w				
HCQ	200 mg/d	200 mg/d	Q2w	200 mg/d	200 mg/d	200 mg/d	200 mg/d	200 mg/d
IFE	IgGγ		200 mg/d					

Abbreviations: ANA, antinuclear antibody; BUN, Blood urea nitrogen; CRP, C‐reactive protein; CTX, cyclophosphamide; ESR, Erythrocyte sedimentation rate; HCQ, hydroxychloroquine sulfate; IFE, immunofixation electrophoresis; MMF, mycophenolate mofetil; PLT, platelet; RBC, Red blood cells; Scr, Serum creatinine; UA, uric acid; WBC, White blood cells.

## 3 LITERATURE REVIEW

Considering that the previous literature review before 2000 was outdated, the Pubmed and Mendeley databases were searched from January 2000 to January 2022 using the following search terms: “systemic lupus erythematosus”; and “multiple myeloma.” The inclusion criteria for the selected articles were as follows: (i) definite diagnosis of SLE and MM; (ii) human studies; and (iii) articles published in English. Articles were excluded for the following reasons: (i) duplicate data were published from the same center; and (ii) reviews or meta‐analyses. Based on the search results, one researcher reviewed the title and the abstract, then two authors confirmed the selected articles and read the full text. The authors analyzed and extracted the data independently and merged the two sets of data into one database. A discussion was arranged to resolve disputed data. The following information and outcome measures were obtained using a predesigned data scheme: author; year of publication; age of SLE onset; age of MM onset; time elapsed to delayed diagnosis of MM; gender; symptoms; diagnostic method; type of MM; treatment regimen for SLE; and MM treatment and evolution.

Thus, relevant studies and cases of SLE with MM were searched within an interval of 22 years. Twelve studies involving 16 cases were preliminarily identified. After eliminating two non‐English studies and three case reports written in Korean and French, plus the two cases reported herein, there were 15 cases of SLE with MM (Table 3).^4^, ^5^, ^6^, ^7^, ^8^, ^9^, ^10^, ^11^, ^12^, ^13^, ^14^

**Figure 1:**
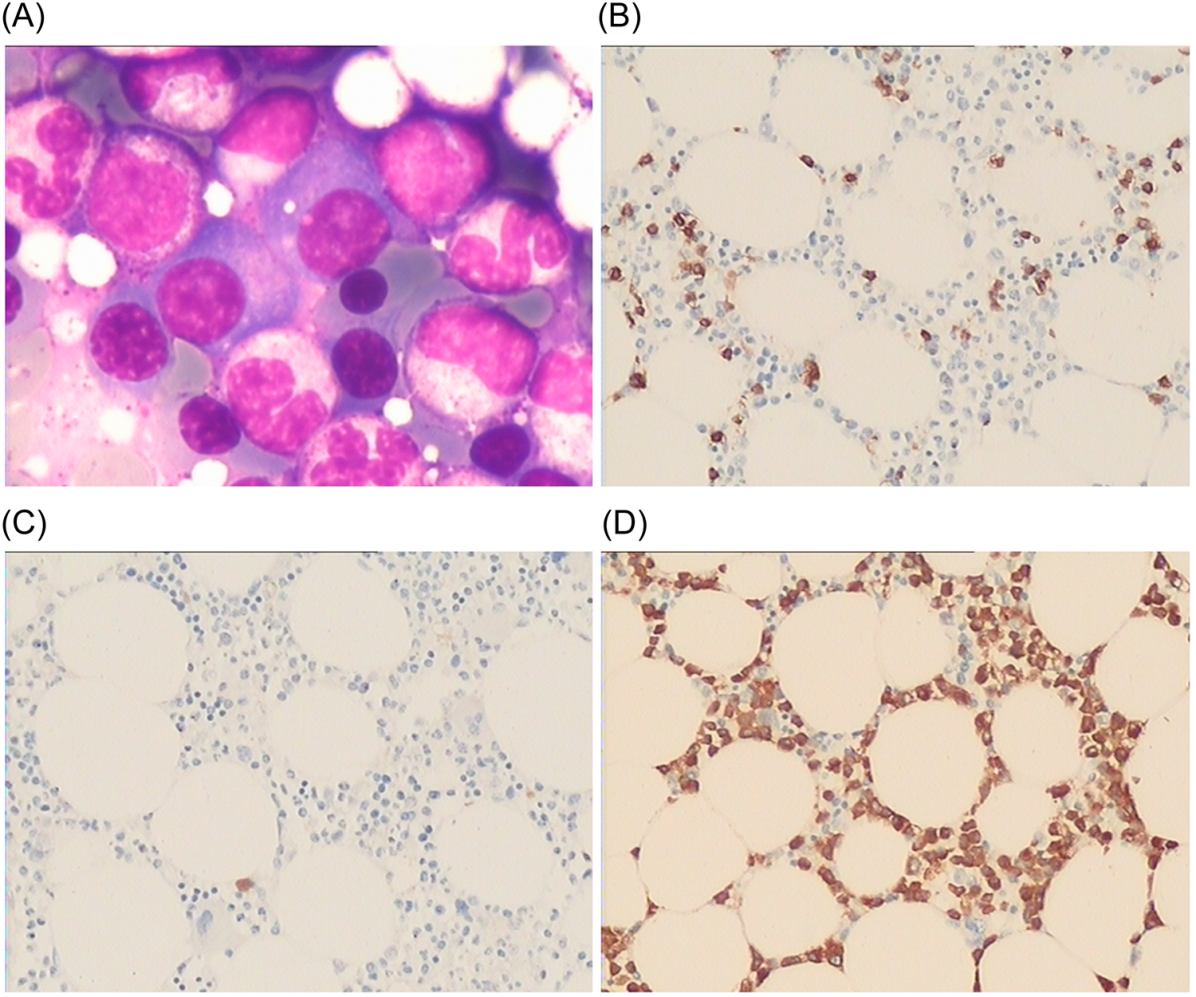
Bone marrow biopsy of Case 1 in this study. (A) HE staining showed that plasma cells increased, accounting for approximately 12% (×400); (B) CD138(+) (×200); (C) Kappa negative (×200); (D) Lambda (+) (×200). Plasma cell myeloma was considered based on the immunohistochemistry results.

**Figure 2:**
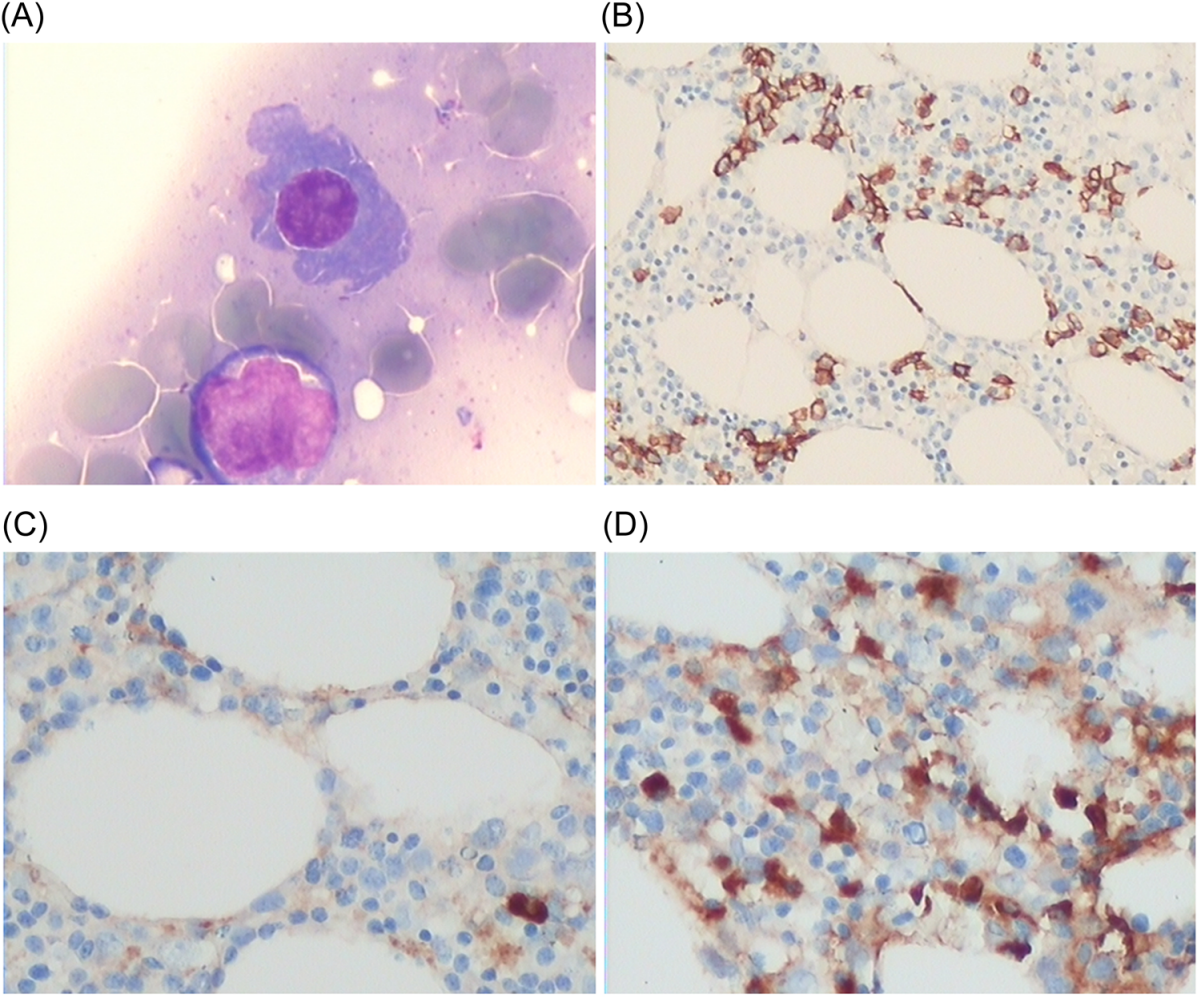
Bone marrow biopsy of Case 2 in this study. (A) HE staining showed increased plasma cells, accounting for approximately 20% (×400); (B) CD138(+) (×200); (C) Kappa negative (×200); (D) Lambda (+) (×200). Plasma cell myeloma was considered based on the immunohistochemistry results.

**Table 3:** Clinical data of SLE associated with MM

Serial no.	Author and publication time (References)	Onset age of SLE	Onset age of MM	Time to delayed diagnosis of MM	Gender	Symptoms	Diagnostic method	Type of MM	Treatment regimen for SLE	MM treatment and evolution
1	Hamza et al.^6^	27‐year‐old	57‐year‐old	30 years	Female, African	Microscopic hematuria, elevated level of protein in urine, quantified as 898 mg/d	Kidney biopsy + bone marrow biopsy + protein electrophoresis	IgG κ	Hormones (15 mg/d) + tacrolimus (4 mg/d)	NA Transferred to other hospital
2	Maamar et al.^4^	43‐year‐old	50‐year‐old	7 years	Female	Pain in the upper left arm, with elevated serum calcium level	Free λ light chains detected by immunofixation electrophoresis in the urine	IgA‐λ	Hormones (60 mg–10 mg/d)	Melphalan + hormones/(VAD); died of septic shock
3	Maamar et al.^4^	32‐year‐old	35‐year‐old	3 years	Female	A sudden elevation of the IgG level with body weight loss and inertia	Protein electrophoresis	IgG‐λ	Hormones (40 mg–10 mg/d) + HCQ (200 mg/d)	Hormones + melphalan, continuous remission during 6 years of follow‐up
4	Okoli et al.^7^	22‐year‐old	28‐year‐old	6 years	Female, African	Abdominal pain, nausea, and body weight loss	Serum protein electrophoresis + immunofixation electrophoresis	IgA κ	HCQ (400 mg/d) + analgesics	Dexamethasone + thalidomide (no follow‐up)
5	Choi et al.^8^	26‐year‐old	31‐year‐old	5 years	Female	Headache, elevated level of protein in the urine	Serum and urine protein electrophoresis + immunoelectrophoresis + bone marrow biopsy	IgA λ	HCQ (400 mg/d)	VAD regimen (VCR + doxorubicin + dexamethasone)/bortezomib + thalidomide + dexamethasone/autologous stem cell transplantation remission
6	Vaiopoulos et al.^9^	76‐year‐old	76‐year‐old	Concomitantly	Female	Skin eruption and hair loss, without typical manifestations of MM	Immunoelectrophoresis + kidney biopsy	IgG‐κ	Hormones + HCQ	Hormones + melphalan
7	Urbańska‐Ryś et al.^10^	38‐year‐old	45	7 years	Female	Combined with myasthenia gravis, keratosis, and enlargement of the left inguinal lymph nodes	Lymph node biopsy + bone marrow hemocytology + protein immunoelectrophoresis	IgGk	Prednisone (60–10 mg/day) + AZP100 mg/day/) CTX 100 mg/day	VAD/CHOP regimen
8	Bila et al.^11^	57‐year‐old	64‐year‐old	7 years	Female	Lumbago and progressive lower extremity weakness	Protein immunoelectrophoresis + bone marrow biopsy	IgA Lambda	Prednisone + HCQ (3 mg/kg/d)	VMCP/VAD regimen/thalidomide + melphalan (having discontinued the treatment for SLE for 2 years); died of pneumonia 4 years later
9	Xu et al.^12^	24‐year‐old	37‐year‐old	13 years	Female	Fracture of the fifth thoracic vertebra after trauma	Kidney biopsy	N/A	NA	Hormones + melphalan; died 6 years later
10	Xu et al.^12^	43‐year‐old	73‐year‐old	30 years	Female	Ostealgia	NA	N/A	Chloroquine phosphate NA	NA (combined with chemotherapy; additional details unknown) died 1 year later
11	Xu and Wiernik ^12^	32‐year‐old	41‐year‐old	9 years	Female	Elevated IgA level in the serum 8 years	Bone marrow biopsy	IgA	Prednisone	No treatment received
12	Kim et al.^13^	55‐year‐old	58‐year‐old	3 years	Female	Anemic proteinuria (MGUS for 3 years), with cutis laxa	Bone marrow biopsy	IgG‐κ	NA	Bortezomib + dexamethasone
13	Wang et al.^5^	47‐year‐old	50‐year‐old	3 years	Female	Chest pain and lumbago, with secondary Sjögren's syndrome	Protein immunoelectrophoresis + bone marrow biopsy	IgG kappa	Prednisone (30 mg–5 mg) + HCQ (400 mg/d) + CTX (800 mg/m)	VAD Died
14	Present study	48‐year‐old	59‐year‐old	11 years	Male	Abnormal kidney function	Immunoprotein electrophoresis + bone marrow biopsy (Figure 1)	IgGλ	Prednisone (60 mg–5 mg/d) + HCQ (400 mg/d) + Cyclosporin (having been discontinued for 4 years)	Bortezomib + dexamethasone + Doxorubicin liposome dexamethasone + lenalidomide Died of septic shock
15	Present study	31‐year‐old	58‐year‐old	27 years	Female	Abnormal kidney function, with proteinuria as a newly occurring symptom	Immunoprotein electrophoresis + bone marrow biopsy (Figure 2)	IgGγ	Prednisone (prednisone 60 mg–10 mg/day) + hydroxychloroquine + cyclophosphamide/mycophenolate mofetil	No treatment received and against advice for MM, discharge with myocardial infarction

Abbreviations: CTX, cyclophosphamide; MGUS, monoclonal gammopathy of undetermined significance; MM, multiple myeloma; SLE, systemic lupus erythematosus.

Among these 15 cases, there were 13 females and 2 males. The average age at the time of SLE diagnosis was 40.07 ± 14.88 years (median age, 38 years; age range, 22–76 years). The average age at the time of MM diagnosis was 50.80 ± 14.62 years (median age, 50 years; age range, 28–76 years). The longest duration of SLE was 30 years (two cases) and the shortest duration was 3 years. The average length of time from SLE‐to‐MM was 10.73 ± 10.02 years (median duration, 7 years; range of duration; 0–30 years). Except for one patient in whom MM and SLE were diagnosed simultaneously, MM occurred after SLE in all other cases. Among the 15 cases, 1 was combined with secondary Sjögren's syndrome,^5^ 1 was combined with myasthenia gravis and keratosis,^11^ and 1 was combined with cutis laxa.^14^ Except for 1 patient in whom SLE and MM were diagnosed simultaneously,^10^ the other 14 patients with SLE were stable. SLE was generally treated by hormones plus hydroxychloroquine, although some patients were also treated with immunosuppressants, including tacrolimus, azathioprine, and CTX.^5^, ^7^, ^11^ One case reported in the present study had discontinued the use of cyclosporin for 4 years and another case was treated with CTX and MMF. In the latter case, proteinuria recurred after discontinuation of immunosuppressants for 10 years. This patient was diagnosed with lupus nephritis, but unresponsive to the treatment with CTX and mycophenolate mofetil. MM primarily presents with microscopic hematuria, proteinuria, ostealgia, bone fractures, lymph node enlargement, an elevated serum calcium level, elevated monoclonal IgG, and abnormal kidney function. Case 11^12^ was only diagnosed with monoclonal gammopathy of undetermined significance (MGUS) alone until confirmation of MM 8 years later. The diagnostic methods for MM include bone marrow biopsy, kidney biopsy, and immunofixation electrophoresis. Except for 2 cases with an unknown type of MM, 8 had IgG‐type MM and 5 had IgA‐type MM. MM was treated with melphalan, thalidomide, and the VAD regimen (V, vincristine; A, Adriamycin; and D, dexamethasone) in these cases. Five patients died (including two in the present study), most often from infections. The Case 1 in the present study died of infectious shock and Case 2 died due to myocardial infarctions.

## 4 DISCUSSION

As reported, the incidence of MM in SLE patients is higher than the general population.^5^ The meta‐analysis by Song^15^ showed that SLE increased the risk of MM (SIR = 1.48, 95% CI = 1.02–2.14); however, the literature search most often revealed sporadic case reports. Based on the literature review, with the exception of one patient in whom SLE and MM were diagnosed simultaneously, MM occurred several years after the diagnosis of SLE in all other patients. We reported herein two patients with SLE complicated by MM (one male and one female) occurring 11 and 27 years after the onset of SLE at 59 and 58 years of age, respectively. These results demonstrated that SLE may increase the risk of MM, and the diagnosis of MM can be missed or MM is easy to misdiagnosis.

The mean median time from the diagnosis of SLE to the diagnosis of MM was 7 years. The median age at the time of diagnosis of MM was 50 years in the present study. Okoli^8^ reported that the median age of SLE with MM was 45 years. In another study, the median age at the time of diagnosis of MM was <64 years of age, which was the median age at the time of diagnosis of MM in the general population.^16^ Our reported cases of SLE were diagnosed with MM at 59 and 58 years of age, respectively. The above facts imply that the genetic or immune factors related to SLE may influence the molecular transformation of MM.^9^

The clinical features of SLE with MM differed from the clinical features of MM alone. Despite the higher incidence of MM in males than females,^17^ 87% (13/15) of the patients with SLE and MM were females, probably due to the fact that SLE predominantly affects females. SLE alone and MM alone are more common in the black population based on previous reports^18^, ^19^; however, only two patients with SLE and MM were black based on our literature review, while all other cases were Caucasians and Asians. SLE with MM does not predominantly affect blacks.

Extramedullary manifestations were more common. The growth and survival of myeloma cells depend on the bone marrow microenvironment. Extramedullary involvement observed in patients with MM alone usually indicates that the patients have progressed to the late stage or patients who have relapsed. Based on our literature review, the incidence of extramedullary involvement was higher in patients with SLE and MM. Among the 15 cases, 8 had extramedullary involvement, accounting for 53.3% (8/15). One study suggested that the high incidence of extramedullary involvement in SLE patients with MM might be related to the early onset of MM.^20^ Extramedullary involvement was observed in 40% of the MM patients <55 years of age at the time of diagnosis.^20^ According to another study, extramedullary involvement is related to activation of the B lymphocyte stimulator (BlyS), which further affects the growth and survival of MM cells.^21^ High BlyS expression in SLE patients has an important impact on the growth and survival of MM cells.^21^ The two patients reported in our study had long‐term stable SLE, and immunosuppressants had been discontinued for many years. The two patients had no obvious bone lytic manifestations, such as bone pain, and both exhibited extramedullary manifestations of abnormal renal function, with decreased complement activity, accompanied by increased monoclonal globulin. Therefore, abnormal renal function was more likely to be misdiagnosed as the disease activity of SLE itself. Moreover, the abnormal renal function in Case 2 lasted 4 years from detection until the diagnosis of MM.

Therefore, when a patient with stable SLE exhibit abnormal renal function that cannot be explained by the primary disease, an elevated monoclonal immunoglobulin level, progressive elevation of one monoclonal globulin and/or associated decline of another clonal globulin, and a decreased ANA titer or even a change from positive to negative, the patient should not be simply treated for an active or secondary manifestation of SLE. Such patients must be examined and screened with immunostationary electrophoresis and bone marrow cells or biopsy to further confirm the diagnosis or diagnose suspected MM as early as possible. In addition, for patients who have already been diagnosed with MM, the presence of other autoimmune diseases, such as SLE, must also be considered due to the underlying correlations.

The potential pathogenesis of lymphoproliferative diseases associated with SLE has not been established; however, the following pathogenic mechanisms have been proposed^22^: B‐cell hyperactivity and defective immune surveillance may help B‐cell clones escape the immune system; increased resistance to cell apoptosis may further promote the malignant transformation of tumors; and mutations in the phosphatase and tensin homolog (PTEN) gene and Bcl‐2 overexpression may be the basis for resistance to cell apoptosis. Among SLE patients, immunosuppressive therapy, continuous Epstein‐Barr virus (EBV) infection, or other concurrent autoimmune diseases may increase the risk of malignancies.^22^, ^23^ Only 3 of 13 cases in the literature review were prescribed immunosuppressants. Both cases reported in the present study received one‐time treatment with CTX/MMF; however, whether the use of cytotoxic drugs in patients with SLE can induce tumors is still unknown. The immune dysfunction associated with SLE has attracted much attention. Studies have shown that suppressor T‐cell dysfunction and functional defects in NK cells may lead to an abnormal proliferative response of B cells to various autoantigens. In patients with SLE, T‐ and B‐cell hyperactivity and continuous activation of B cells may be conducive to the appearance of abnormal or malignant plasma cell clones. The incidence of rheumatoid arthritis and SLE is higher in first‐degree relatives of MM patients, thus indicating the effects of genetic factors. Landgren et al.^24^ also reported an increased risk of MM in those with a family history of SLE (OR, 2.66; 95% CI, 1.12–6.32). The aunt of case number 11 died of MM at 50 years of age but had no history of SLE. None of the other patients had a family history of SLE or MM. The two cases reported in the present study had no genetic background or family history of SLE and MM. The heredity of SLE with MM is still waiting for further elucidation.

Currently, there are no specific guidelines for the treatment of SLE with MM. Because the conditions of cases in the previous reports and Case 1 in our report were stable with respect to SLE, the patients primarily received treatment for MM using the VAD regimen. Case 2 reported herein was once misdiagnosed with abnormal kidney function and proteinuria related to SLE. Therefore, the treatment targeting SLE was strengthened in this case; however, CTX was no longer used after the diagnosis of MM was made.

## 5 SUMMARY

The diagnosis of MM is easily delayed or misdiagnosed in patients diagnosed with SLE. In stable SLE patients with extramedullary involvement, abnormal kidney function or an elevation of a monoclonal immunoglobulin level, SLE complicated with MM may be suspected. These patients must be examined and screened with immunostationary electrophoresis and bone marrow cells or biopsy to further confirm the diagnosis or diagnose suspected MM as early as possible.

## CONFLICT OF INTEREST

The authors declare no conflict of interest.

## Data Availability

The data that support the findings of this study are available from the corresponding author upon reasonable request.
